# Predictors and Outcomes of In-Hospital Cardiac Arrest in University Medicine with Advanced Tertiary and Transplant Care

**DOI:** 10.3390/medsci14040480

**Published:** 2026-08-14

**Authors:** Mohamad Amer Nashtar, Patrick Hjalmar Nekarda, Varnavas Varnavas, Asterios Tzalavras, Jan Best, Ali Canbay, Tim Rahmel, Jordi Rello, Antonios Katsounas

**Affiliations:** 1 Ruhr University Bochum, Knappschaft Kliniken University Hospital Bochum, Department of Medicine, 44892 Bochum, Germany; mohamad.nashtar@knappschaft-kliniken.de (M.A.N.); patrick.nekarda@knappschaft-kliniken.de (P.H.N.); asterios.tzalavras@knappschaft-kliniken.de (A.T.); ali.canbay@rub.de (A.C.); antonios.katsounas@rub.de (A.K.); 2 Division of Cardiology, Department of Cardiovascular Diseases, Cliniques Universitaires St. Luc, 1200 Brussels, Belgium; varnavas.varnavas@saintluc.uclouvain.be; 3 Department of Gastroenterology, Hepatology, and Transplant Medicine, University Hospital Essen, University of Duisburg Essen, 45147 Essen, Germany; jan.best@uk-essen.de; 4 Ruhr University Bochum, Knappschaft Kliniken University Hospital Bochum, Department of Anesthesiology, Intensive Care and Pain Therapy, 44892 Bochum, Germany; tim.rahmel@rub.de; 5 Department of Medicine, Faculty of Medicine and Health Sciences, Universitat Internacional de Catalunya, 08195 Barcelona, Spain; 6 Clinical Research/Innovation in Pneumonia & Sepsis (CRIPS), Vall d’Hebron Research Institute (VHIR), 08035 Barcelona, Spain; 7 Centro de Investigación Biomédica en Red de Enfermedades Respiratorias (CIBERES), Instituto de Salud Carlos III, 28029 Madrid, Spain; 8 IMAGINE, UR-UM 107, University of Montpellier, Division of Anaesthesia Critical Care, Pain and Emergency Medicine, Nîmes University Hospital, 30029 Nîmes, France

**Keywords:** in-hospital cardiac arrest, cardiopulmonary resuscitation, return of spontaneous circulation, non-shockable rhythm, reversible causes, survival, prognostic factors

## Abstract

Background/Objectives: In-hospital cardiac arrest (IHCA) remains associated with poor survival, while the contribution of arrest etiology and early physiological markers to outcome remains incompletely defined. We examined associations between clinical, neurological, metabolic, and etiological factors and sustained return of spontaneous circulation (ROSC) lasting >20 min, 28-day survival, and 60-day mortality. Methods: This retrospective single-center cohort included 134 adults with confirmed IHCA requiring cardiopulmonary resuscitation at a tertiary university hospital with advanced transplant care between 2011 and 2015. Sustained ROSC and 28-day survival were analyzed using parsimonious Firth penalized logistic regression models, and 60-day mortality using Cox proportional hazards regression. Selected neurological and biomarker analyses were considered exploratory. Results: Sustained ROSC was achieved in 91 patients (67.9%), 33 (24.6%) survived to day 28, and 30 (22.4%) survived to day 60. Initial non-shockable rhythm was associated with lower odds of sustained ROSC (aOR 0.26, 95% CI 0.09–0.69) and 28-day survival (aOR 0.27, 95% CI 0.10–0.68), and with higher 60-day mortality (aHR 1.96, 95% CI 1.25–3.08). The presence of a potentially reversible cause was associated with more favorable outcomes, whereas active hematologic malignancy was associated with higher 60-day mortality (aHR 1.89, 95% CI 1.15–3.10). Exploratory analyses also showed associations of initially dilated pupils with lower 28-day survival and higher lactate with 60-day mortality. Among patients who survived beyond 24 h, higher BNP was associated with subsequent mortality. Conclusions: Non-shockable rhythm and the presence of a potentially reversible cause were consistently associated with outcomes following IHCA, while active hematologic malignancy was associated with poorer longer-term survival. Exploratory findings from lactate, initial pupillary assessment, and BNP may provide complementary prognostic information, although the BNP analysis was limited to patients who survived beyond 24 h. These findings require confirmation in contemporary multicenter cohorts and do not establish validated clinical prediction tools.

## 1. Introduction

In-hospital cardiac arrest (IHCA) remains a major global healthcare challenge, with persistently high morbidity and mortality despite advances in resuscitation science and post–cardiac arrest care. Current estimates indicate that approximately 290,000 IHCAs occur annually in the United States, while in Europe the annual incidence ranges between 1.5 and 2.8 per 1000 hospital admissions, with survival rates varying widely across institutions and health systems [1,2]. Although outcomes have gradually improved, overall survival to hospital discharge remains limited, ranging from 15% to 25%, and only a minority of patients achieve favorable neurological outcomes [3,4].

The prognostic determinants of survival after IHCA are multifactorial and encompass pre-arrest comorbidities, arrest characteristics, and post-resuscitation physiological markers. Large registry-based studies from the American Heart Association Get With The Guidelines–Resuscitation (AHA GWTG-R) program have consistently demonstrated that initial rhythm, timeliness of cardiopulmonary resuscitation (CPR), witnessed arrest, and location of the arrest are associated with the likelihood of return of spontaneous circulation (ROSC) and longer-term survival [4,5,6,7]. Notably, patients with initial shockable rhythms (ventricular fibrillation, VF/pulseless ventricular tachycardia, VT) show significantly better outcomes compared with those presenting with asystole or pulseless electrical activity (PEA) [4].

Beyond clinical arrest features, metabolic and cardiovascular biomarkers, including lactate and B-type natriuretic peptide (BNP), have been associated with post-arrest outcomes, although their incremental prognostic value and optimal clinical use remain incompletely defined [8,9].

In addition to patient-specific factors, the etiology of cardiac arrest represents a central determinant of the in-hospital resuscitation context. Arrests resulting from potentially reversible causes—such as acute pulmonary embolism (PE), electrolyte disturbances, or hypoxia—constitute a distinct clinical category in which timely recognition and targeted interventions may influence the immediate resuscitation course [10,11,12,13].

Despite notable advances, available IHCA risk-stratification tools do not consistently incorporate arrest etiology, patient-specific characteristics, or early metabolic markers, and their performance may vary across clinical settings. We therefore conducted a retrospective cohort study at a tertiary academic medical center to examine associations between clinical, metabolic, and etiological factors and post-resuscitation outcomes. The study was designed to provide hypothesis-generating evidence rather than to develop or externally validate a prognostic score. This investigation forms part of a broader institutional analysis of code blue activations; a companion study focusing on non-arrest events has been published previously [14].

## 2. Methods

### 2.1. Study Design and Patient Selection

This retrospective observational study was conducted at the University Hospital Essen, Germany, a tertiary academic care center with approximately 1300 inpatient beds.

Medical records and institutional resuscitation protocols were reviewed for all IHCA events with documented CPR between January 2011 and February 2015. Records from the calendar year 2012 could not be included because the original paper-based resuscitation protocols were not retrievable during cohort assembly owing to a disruption of archival continuity associated with an institutional relocation in 2014. The omission was therefore administrative and was not based on patient characteristics, arrest features, or clinical outcomes. This analysis represents a predefined sub-cohort of a comprehensive institutional evaluation of code blue activations; detailed characteristics of the overall cohort and study framework have been published previously [14].

A total of 134 patients with sufficiently complete resuscitation documentation and available outcome data were included. Eligible patients were aged ≥18 years and had a confirmed cardiac arrest requiring CPR, verified by the documented clinical presentation and records of the in-hospital resuscitation team. Patients were excluded if no true cardiac arrest was present, e.g., pseudo-PEA or syncope misclassified as arrest, if the available documentation was insufficient to confirm the arrest, or if post-resuscitation outcome data were unavailable. The rhythm at CPR-team arrival was defined as the first rhythm documented by the in-hospital resuscitation team and did not necessarily represent the rhythm at cardiac arrest onset. In 14 patients, CPR had been initiated and ROSC achieved before the team’s first rhythm assessment. Sinus rhythm was documented in 11 patients, whereas no rhythm was documented in three. Because the preceding arrest rhythm was unavailable and no defibrillation had been recorded, these patients could not be reliably classified as having a shockable or non-shockable arrest rhythm and were therefore excluded from direct comparisons between shockable and non-shockable rhythm groups.

For each included case, demographic characteristics, comorbidities, arrest circum-stances, resuscitation measures, laboratory values, and outcomes were abstracted. The primary outcomes were sustained ROSC lasting > 20 min, survival to day 28, and all-cause mortality through day 60. Any ROSC and 48-h survival were summarized descriptively as secondary outcomes. For analyses of 60-day mortality, follow-up began at CPR onset; patients alive at day 60 were administratively censored.

### 2.2. Statistical Methods

All data were extracted from the hospital information system and institutional resuscitation protocols and subsequently transferred to Microsoft Excel for preprocessing and quality control. Statistical analyses were performed using GraphPad Prism (version 10.6; GraphPad Software, San Diego, CA, USA) and Python (version 3.11; Python Software Foundation, Wilmington, DE, USA).

Distributional assumptions were assessed using histograms, Q–Q plots, and the Shapiro–Wilk test. Continuous variables were summarized as mean ± standard deviation (SD) when approximately normally distributed and as median with interquartile range (Q1–Q3) otherwise. Categorical variables were presented as absolute frequencies and percentages. Between-group comparisons used Welch’s *t*-test or the Mann–Whitney U test for continuous variables, as appropriate. Categorical variables were compared using Pear-son’s chi-square test without continuity correction when all expected cell counts were ≥5; otherwise, Fisher’s exact test was used. These comparisons were descriptive, and *p*-values were not adjusted for multiple testing. Time-to-event outcomes were analyzed using Kaplan–Meier methods, with group differences assessed by the Log-rank (Mantel–Cox) test.

To limit overfitting and model proliferation, multivariable analyses were restricted to sustained ROSC lasting > 20 min, 28-day survival, and 60-day mortality. Covariates were selected on clinical grounds and with attention to model parsimony; univariable *p*-value screening and automated stepwise selection were not used. Sustained ROSC and 28-day survival were analyzed using Firth’s bias-reduced penalized-likelihood logistic regression. The sustained-ROSC model included age per 10-year increase, documented non-shockable rhythm at CPR-team arrival, arrest witnessed by medical staff, and any reversible cause of cardiac arrest. The 28-day survival model included age, documented non-shockable rhythm, and any reversible cause. Odds ratios were reported with profile penalized-likelihood 95% confidence intervals, and *p*-values were based on penalized likelihood-ratio tests.

Mortality through day 60 was analyzed using Cox proportional hazards regression with Efron handling of tied event times. The primary Cox model included age per 10-year increase, documented non-shockable rhythm at CPR-team arrival, any reversible cause, and active hematologic malignancy. Hazard ratios were reported with Wald 95% confidence intervals and Wald *p*-values. The proportional-hazards assumption was assessed using scaled Schoenfeld residuals, and the functional form of continuous covariates was examined in sensitivity analyses. The aggregate variable “any reversible cause” comprised hypoxia, hypovolemia, hypothermia, hypo-/hyperkalemia, or PE, with each patient counted once.

Model performance was assessed using the area under the receiver operating characteristic curve (AUC) for logistic models, Harrell’s C-index for Cox models, and Brier scores. Apparent and optimism-corrected estimates were obtained by bootstrap internal validation with 1000 resamples. Apparent 95% confidence intervals were based on bootstrap percentiles, whereas optimism-corrected intervals were derived using a location-shifted bootstrap approach. These estimates reflect internal model performance and do not constitute external validation.

Targeted exploratory analyses addressed selected clinical questions. Active hematologic malignancy and initially dilated pupils at CPR-team arrival were evaluated in separate extensions of the 28-day survival model. Lactate was examined continuously in a complete-case Cox analysis of 60-day mortality among patients with available measurements at CPR-team arrival. BNP was evaluated using a 24-h landmark Cox analysis restricted to patients who survived beyond 24 h and had a BNP measurement within that period; BNP was log_2_-transformed, and hazard ratios were expressed per doubling. PE-related outcomes were summarized in an unadjusted exploratory analysis using Fisher’s exact test and absolute risk differences with 95% Newcombe–Wilson confidence intervals. A lactate threshold for 60-day mortality was derived using the Youden index and used only for exploratory Kaplan–Meier visualization. Because this threshold was derived and evaluated in the same cohort, it was not considered independently validated. Missing data were not imputed, and targeted secondary and exploratory analyses were not adjusted for multiplicity. All tests were two-sided, with *p* < 0.05 considered statistically significant.

## 3. Results

### 3.1. Patient Characteristics

A total of 134 patients with confirmed cardiac arrest and documented CPR were included in the analysis. The mean age of the cohort was 64 ± 14 years, and the majority were male (67.9%). Regarding resuscitation characteristics, the median duration of CPR was 45 min (30–61). Patients without ROSC more frequently exhibited initially dilated pupils at CPR-team arrival (57.1% vs. 32.3%, *p* = 0.0096). The total epinephrine dose administered was significantly higher in non-ROSC patients (7 mg; 4.3–11.5) compared with those achieving ROSC (3 mg; 1–5, *p* < 0.0001). Rocuronium use was more common in patients with ROSC (45.5% vs. 20.0%, *p* = 0.0079). Moreover, cardiac arrests witnessed by medical staff were more frequent in the ROSC group (75.8% vs. 51.4%, *p* = 0.0073).

With respect to initial rhythm, non-shockable rhythms (asystole or PEA) were significantly more frequent in the non-ROSC group (88.6% vs. 62.6%, *p* = 0.0042), whereas initial shockable rhythms (VF or VT) were more common in patients achieving ROSC (24.2% vs. 8.6%, *p* = 0.0470).

PE was more frequently documented among patients who achieved ROSC than among those who did not (22.2% vs. 2.9%, *p* = 0.0081). The derived aggregate category “any reversible cause,” comprising hypoxia, hypovolemia, hypothermia, hypo-/hyperkalemia, and PE, was also significantly more frequent in the ROSC group (42.4% vs. 20.0%, *p* = 0.0179), with PE representing the most frequently documented component. An unclear or multifactorial etiology was numerically more frequent among patients without ROSC, although this difference did not reach statistical significance (22.9% vs. 11.1%, *p* = 0.0974).

At the descriptive level, liver cirrhosis was more frequent among patients who achieved ROSC than among those who did not (25.3% vs. 8.6%, *p* = 0.0369). No significant differences were observed for other chronic conditions, including coronary artery disease, hypertension, diabetes mellitus, chronic kidney disease, or malignancy.

Laboratory parameters at CPR-team arrival showed no statistically significant differences between groups. Median lactate and hemoglobin levels were comparable, as were serum potassium and glucose levels.

Overall, patients achieving ROSC were more likely to have a witnessed arrest, a shockable rhythm and lower cumulative epinephrine doses, while non-ROSC patients more frequently presented with initially dilated pupils, non-shockable rhythms, and unclear or multifactorial etiologies. A detailed overview of baseline characteristics and statistical comparisons is presented in Table 1.

### 3.2. Factors Associated with Sustained ROSC (>20 Min)

A non-shockable rhythm at CPR-team arrival remained associated with lower odds of sustained ROSC after adjustment, whereas the presence of any reversible cause was associated with higher odds. Witnessed arrest showed a positive but non-significant association after adjustment, while age was not associated with the outcome (Table 2). The multivariable model was statistically significant overall (penalized likelihood-ratio χ^2^ = 22.03, df = 4, *p* = 0.0002) and showed moderate discrimination, with an apparent AUC of 0.741 (95% bootstrap CI 0.668–0.837) and an optimism-corrected AUC of 0.711 (95% location-shifted bootstrap CI 0.639–0.807) after 1000 bootstrap resamples. The apparent and optimism-corrected Brier scores were 0.184 and 0.199, respectively.

### 3.3. Factors Associated with 28-Day Survival After In-Hospital Cardiac Arrest

A non-shockable rhythm at CPR-team arrival remained associated with lower odds of 28-day survival after adjustment, whereas the presence of any reversible cause was associated with higher odds. Age was not associated with the outcome (Table 3). The multivariable model was statistically significant overall (penalized likelihood-ratio χ^2^ = 43.81, df = 3, *p* < 0.0001) and showed good discrimination, with an apparent AUC of 0.841 (95% bootstrap CI 0.764–0.906) and an optimism-corrected AUC of 0.829 (95% location-shifted bootstrap CI 0.752–0.893) after 1000 bootstrap resamples. The apparent and optimism-corrected Brier scores were 0.137 and 0.148, respectively.

In targeted secondary analyses, active hematologic malignancy remained associated with lower odds of 28-day survival after adjustment for age, non-shockable rhythm, and the presence of any reversible cause (adjusted OR = 0.18, 95% CI 0.02–0.91, *p* = 0.0367). In a separate extension model, initially dilated pupils at CPR-team arrival were also associated with substantially lower odds of 28-day survival (adjusted OR = 0.09, 95% CI 0.01–0.33, *p* = 0.0001). The associations of non-shockable rhythm and any reversible cause remained directionally consistent in both models. These analyses were exploratory and are presented in Supplementary Table S1.

### 3.4. Factors Associated with 60-Day Mortality After In-Hospital Cardiac Arrest

A non-shockable rhythm at CPR-team arrival and active hematologic malignancy remained associated with a higher hazard of 60-day mortality after adjustment, whereas the presence of any reversible cause was associated with a lower mortality hazard. Age was not associated with the outcome (Table 4). The multivariable model was statistically significant overall (likelihood-ratio χ^2^ = 52.09, df = 4, *p* < 0.0001) and showed moderate discrimination, with an apparent Harrell C-index of 0.752 (95% bootstrap CI 0.697–0.810) and an optimism-corrected C-index of 0.745 (95% location-shifted bootstrap CI 0.689–0.803) after 1000 bootstrap resamples. The apparent and optimism-corrected 60-day Brier scores were 0.120 and 0.129, respectively.

**Figure 1:**
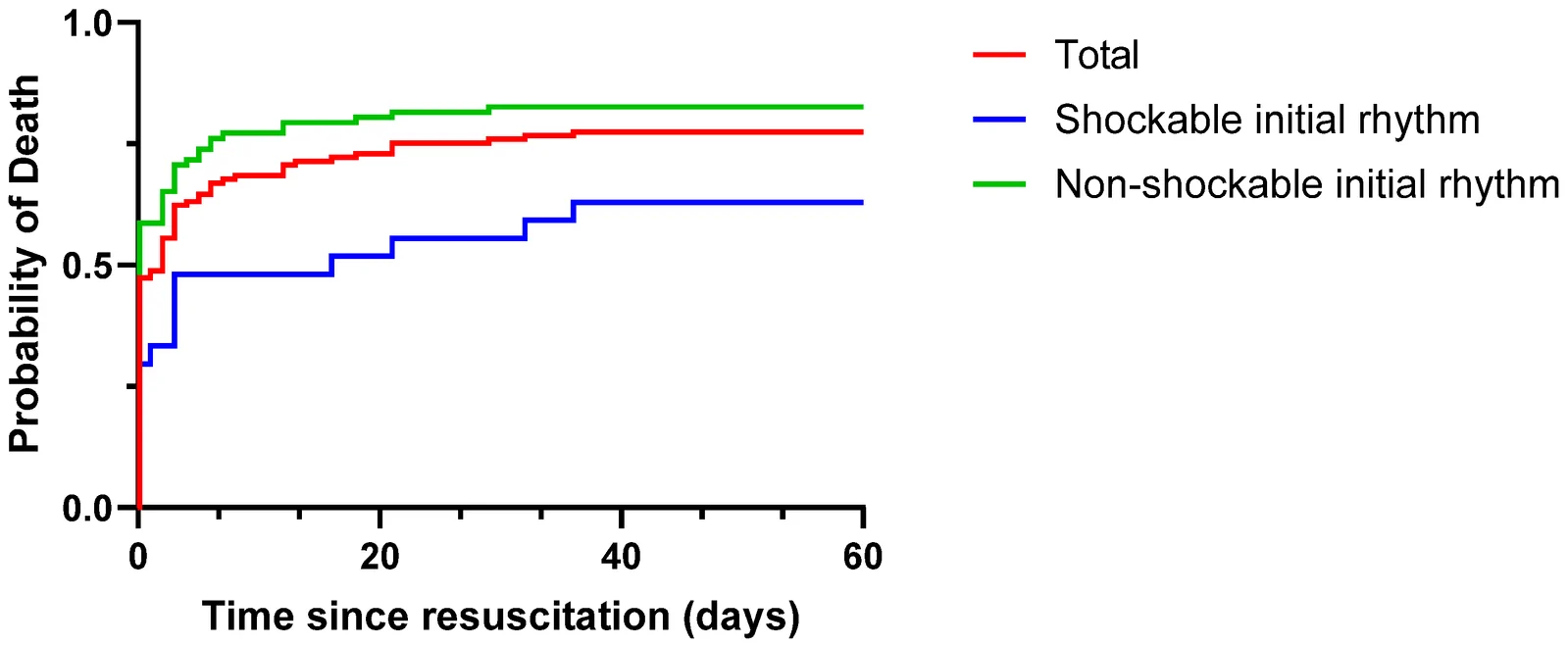
Kaplan–Meier estimates of cumulative 60-day mortality after in-hospital cardiac arrest, stratified by initial rhythm at CPR-team arrival. The curves depict the cumulative probability of death, calculated as 1 minus the Kaplan–Meier survival estimate, from the onset of cardiopulmonary resuscitation through day 60. The red curve represents the entire cohort (*n* = 134) and is shown for descriptive reference. For the stratified analysis, patients with a shockable initial rhythm (ventricular fibrillation or pulseless ventricular tachycardia; *n* = 27) were compared with those with a non-shockable initial rhythm (asystole or pulseless electrical activity; *n* = 93). Patients with sinus rhythm (*n* = 11) or no documented rhythm (*n* = 3) at CPR-team arrival had achieved ROSC before the team’s first rhythm assessment and were excluded because the arrest rhythm could not be classified as shockable or non-shockable. Differences between the two rhythm groups were assessed using the two-sided log-rank (Mantel–Cox) test (*p* = 0.0421).

Consistent with the Cox regression findings, cumulative 60-day mortality was higher among patients with a non-shockable initial rhythm than among those with a shockable initial rhythm at CPR-team arrival (log-rank *p* = 0.0421; Figure 1).

**Figure 2:**
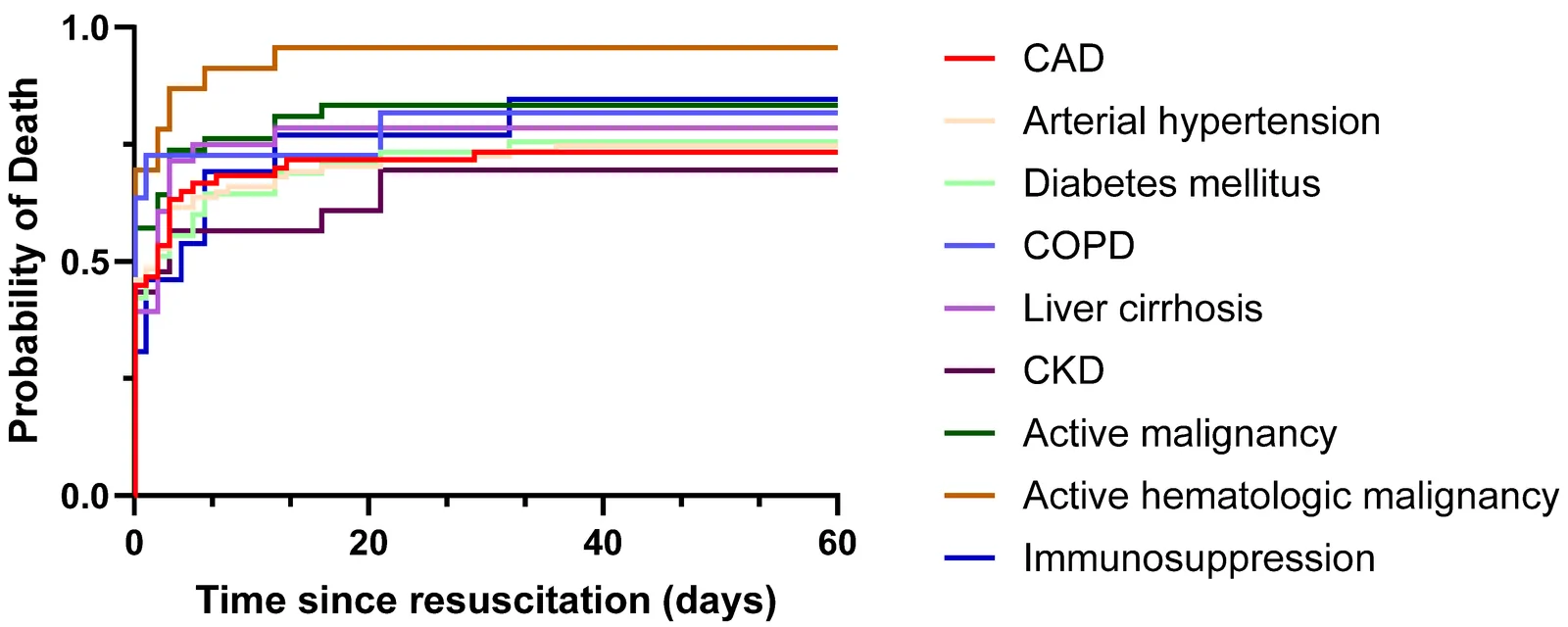
Kaplan–Meier estimates of cumulative 60-day mortality after in-hospital cardiac arrest, stratified by comorbidities. The curves depict the cumulative probability of death, calculated as 1 minus the Kaplan–Meier survival estimate, from the onset of cardiopulmonary resuscitation through day 60. Patients with active hematologic malignancy showed significantly higher mortality compared to those without this condition (*p* = 0.0017). Comorbidity categories were not mutually exclusive; curves for comorbidities other than active hematologic malignancy are presented for descriptive purposes only. Group sizes were as follows: coronary artery disease, *n* = 61; arterial hypertension, *n* = 92; diabetes mellitus, *n* = 45; chronic obstructive pulmonary disease, *n* = 11; liver cirrhosis, *n* = 28; chronic kidney disease, *n* = 24; active malignancy, *n* = 42; active hematologic malignancy, *n* = 23; and immunosuppression, *n* = 13. Abbreviations: CAD = coronary artery disease; COPD = chronic obstructive pulmonary disease; CKD = chronic kidney disease.

Among the comorbidities examined, active hematologic malignancy was associated with higher cumulative 60-day mortality compared with its absence (log-rank *p* = 0.0017), whereas no clear associations were observed for the other individual comorbidities in exploratory unadjusted comparisons. Active hematologic malignancies were heterogeneous, comprising predominantly low-grade lymphoid malignancies (73.9%), followed by acute leukemias (17.4%) and high-grade lymphomas (8.7%). This observation further supports the prognostic impact of active hematologic malignancy on post-resuscitation survival outcomes (Figure 2).

In a complete-case secondary analysis of patients with available lactate measurements (*n* = 91; 65 deaths), higher lactate concentrations at CPR-team arrival remained associated with a higher hazard of 60-day mortality after adjustment for age, non-shockable rhythm, any reversible cause, and active hematologic malignancy. The model showed good discrimination after bootstrap correction. Detailed regression estimates and model performance are provided in Supplementary Table S2.

For descriptive purposes, receiver operating characteristic (ROC) analysis identified an exploratory lactate threshold of 3.9 mmol/L for 60-day mortality using the Youden index (sensitivity 87.7%, specificity 73.1%). In an exploratory Kaplan–Meier analysis, patients with lactate concentrations ≥ 3.9 mmol/L had higher cumulative 60-day mortality than those with concentrations < 3.9 mmol/L (log-rank *p* < 0.0001; Supplementary Figure S1). Because the threshold was derived and evaluated in the same cohort, it should not be considered independently validated.

BNP was measured within 24 h after resuscitation in 71 patients. The median BNP concentration was 443.7 pg/mL (132.0–1080.0), and the median interval from the end of resuscitation to blood sampling was 10 h (6–16). The targeted 24-h landmark analysis included 55 patients who survived beyond 24 h and had an available BNP measurement; the median BNP concentration in this landmark cohort was 257.6 pg/mL (128.8–1080.0). Among these patients, 31 subsequently died before day 60. Each doubling of BNP was associated with a higher hazard of subsequent mortality after adjustment for age, non-shockable rhythm, any reversible cause, and active hematologic malignancy (adjusted HR = 1.31, 95% CI 1.09–1.59, *p* = 0.0049). Detailed regression estimates and internally validated model performance measures are provided in Supplementary Table S3.

### 3.5. Secondary and Exploratory Outcomes

Any ROSC was achieved in 99 of 134 patients (73.9%), and 69 patients (51.5%) survived for at least 48 h. These secondary outcomes were reported descriptively to avoid redundant endpoint-specific modeling. In exploratory unadjusted analyses, outcome rates were consistently higher in PE-related IHCA; detailed event rates and absolute risk differences are provided in Supplementary Table S4. Given the small PE subgroup and near-complete separation of several outcomes, PE was not included as an individual predictor in the multivariable models.

## 4. Discussion

In this retrospective cohort of patients with in-hospital cardiac arrest, documented non-shockable rhythm at CPR-team arrival was associated with less favorable outcomes, whereas the presence of at least one potentially reversible cause was associated with higher odds of sustained ROSC and 28-day survival and a lower hazard of 60-day mortality. Active hematologic malignancy was associated with poorer longer-term survival. Targeted exploratory analyses additionally identified associations of initially dilated pupils with 28-day survival, lactate at CPR-team arrival with 60-day mortality, and BNP measured within 24 h with subsequent mortality. These findings illustrate the multifactorial nature of IHCA outcomes but should be interpreted as hypothesis-generating associations rather than as evidence of causal effects or a validated prognostic model.

The prognostic relevance of the initial rhythm was also evident in our analysis. An initial non-shockable rhythm (asystole/PEA) at CPR-team arrival remained associated with lower odds of sustained ROSC and 28-day survival and with a higher hazard of 60-day mortality after adjustment. This finding is consistent with large multicenter data from the AHA GWTG-R registry and other studies demonstrating substantially poorer survival among patients presenting with an initial non-shockable rhythm compared with those with a shockable rhythm [1,3,4,15]. These differences may partly reflect variation in arrest etiology, underlying patient characteristics, and the absence of an immediately defibrillation-responsive rhythm [1,15].

The presence of at least one potentially reversible cause was consistently associated with more favorable outcomes. This finding supports the clinical importance of promptly identifying and treating reversible causes during resuscitation, as emphasized in current European Resuscitation Council (ERC) and American Heart Association (AHA) recommendations [10,11]. However, this aggregate combined etiologically distinct conditions and should not be interpreted as implying a uniform prognostic effect across all reversible causes. Because PE accounted for 23 of the 49 patients in this category and showed particularly favorable unadjusted outcomes, the overall association may have been disproportionately influenced by the PE subgroup. The small numbers within the remaining etiologic categories and near-complete separation for PE precluded reliable cause-specific multivariable analyses. The PE-related pattern should therefore not be interpreted as an independent protective effect and may reflect patient selection, early recognition, cause-directed treatment, or unmeasured differences in arrest circumstances. Although interventions such as systemic thrombolysis, catheter-based reperfusion, and extracorporeal support may improve outcomes in selected patients with PE-related cardiac arrest, survival remains highly dependent on clinical context, patient selection, and timely access to treatment [12,13,16,17]. All patients with PE-related IHCA in our cohort received systemic thrombolysis during resuscitation. However, thrombus age and the exact timing of thrombolytic treatment could not be reliably reconstructed, and no untreated comparison group was available. The unusually favorable outcome pattern therefore cannot be attributed to early thrombosis or to thrombolysis itself.

Initially dilated pupils at CPR-team arrival were associated with markedly lower odds of 28-day survival in a targeted exploratory analysis. This early observation concerned pupillary size rather than a standardized assessment of pupillary light reactivity and should not be equated with delayed multimodal neurological prognostication after cardiac arrest [18,19,20,21]. Initial pupillary dilation may reflect severe cerebral hypoperfusion or prolonged circulatory arrest, but non-standardized assessment, residual confounding, and possible influence on treatment decisions cannot be excluded. The finding should therefore be considered exploratory and requires confirmation using standardized neurological examinations.

Higher lactate at CPR-team arrival remained associated with a higher hazard of 60-day mortality in the complete-case biomarker analysis. Lactate is an established marker of global tissue hypoperfusion and the severity of ischemia–reperfusion injury after cardiac arrest [8,22]. Nevertheless, a single measurement may also be influenced by arrest duration, epinephrine administration, hepatic or renal dysfunction, and the underlying cause of arrest. The ROC-derived threshold of 3.9 mmol/L provided descriptive separation of cumulative 60-day mortality but was derived and evaluated in the same cohort. It should therefore not be interpreted as a clinically validated decision threshold.

In the 24-h landmark analysis, each doubling of BNP was associated with a higher hazard of subsequent mortality through day 60. BNP concentrations were measured at variable times within the first 24 h after resuscitation, with a median sampling time of 10 h (6–16). During this dynamic period, BNP levels may have been influenced by fluid administration, changing renal function, acute ventricular strain, pre-existing cardiac dysfunction, and post-resuscitation myocardial dysfunction. A single measurement obtained within this interval should therefore not be interpreted as a standardized biomarker assessment. Previous studies have reported associations of BNP or NT-proBNP with adverse outcomes after cardiac arrest, although their incremental prognostic value remains incompletely established [9,23]. Because the analysis was restricted to 24-h survivors with an available BNP measurement, the findings apply only to this selected population and not to mortality from the onset of resuscitation. The small landmark cohort, variable sampling time, and selective BNP testing support a cautious, exploratory interpretation.

Active hematologic malignancy was associated with lower odds of 28-day survival in a targeted extension model and with a higher hazard of 60-day mortality in the primary Cox analysis. This is consistent with reports of poor sustained survival after CPR among patients with hematologic malignancies and may reflect the combined effects of active disease, treatment-related complications, immune dysfunction, organ failure, and reduced physiological reserve [24,25]. However, the subgroup was small and clinically heterogeneous, comprising several malignancy types. These results should therefore support individualized clinical assessment rather than categorical decisions regarding resuscitation or post-resuscitation care.

Liver cirrhosis was more frequent among patients who achieved ROSC in the descriptive comparison. All patients fulfilled the same criteria for confirmed cardiac arrest, and pseudo-PEA was excluded; the observed difference is therefore unlikely to be explained by misclassification of profound circulatory collapse as cardiac arrest. Given the small subgroup, multiple descriptive comparisons, and absence of a consistent association with later survival, the finding may reflect residual confounding, unmeasured differences in arrest circumstances or clinical setting, or chance. In-hospital mortality among patients with cirrhosis nevertheless remained high at 78.6%, consistent with previous reports of poor outcomes after in-hospital CPR in this population [26,27]. This finding illustrates that achievement of ROSC does not necessarily translate into sustained survival.

Several additional descriptive findings—including lower cumulative epinephrine dose, more frequent rocuronium use, and a higher proportion of witnessed arrests among patients achieving ROSC—should not be interpreted as treatment effects. Epinephrine exposure and the use of neuromuscular blockade are influenced by arrest duration, severity, airway-management requirements, and clinical circumstances, creating substantial potential for confounding by indication and reverse causation [28,29,30]. Witnessed arrest may similarly reflect a shorter no-flow interval and earlier initiation of CPR. In the sustained-ROSC model, its favorable association was attenuated after adjustment.

Taken together, these findings support the clinical relevance of rhythm assessment, the identification of potentially reversible causes, and early physiological characterization following IHCA. Lactate, BNP, and pupillary findings may provide complementary prognostic information; however, the present analyses do not establish clinically validated thresholds or prediction tools suitable for individual treatment decisions. Prospective multicenter studies with standardized data collection are needed to confirm these associations and to develop and externally validate risk-stratification models incorporating these variables.

### 4.1. Strengths and Limitations

A strength of this study is the comprehensive characterization of arrest circumstances, presumed etiologies, comorbidities, early laboratory findings, and clinically relevant outcomes in a tertiary academic setting. The analytical strategy was deliberately parsimonious and clearly distinguished primary from targeted exploratory analyses, thereby reducing the risk of overfitting and unnecessary multiplicity. In addition, bootstrap-based internal validation provided a more robust assessment of model performance within this modest cohort.

However, several limitations must be acknowledged. The retrospective, single-center design limits external generalizability and may reflect institution-specific practices. In addition, the cohort was not fully consecutive because the paper-based resuscitation records from 2012 could not be retrieved during cohort assembly following a disruption of archival continuity associated with an institutional relocation. Although this omission was administrative and unrelated to known clinical characteristics or outcomes, it may have introduced temporal selection bias and limits the completeness of the cohort. An additional limitation is the historical study period. The cohort reflects resuscitation and post-resuscitation practice from 2011 to 2015, whereas several aspects of contemporary care have subsequently evolved, including temperature-control strategies, cause-directed treatment, neuroprognostication, and the availability and selection of patients for extracorporeal cardiopulmonary resuscitation [11,18,19]. Consequently, absolute outcome rates and treatment-related associations may not be directly transferable to current clinical practice. Although associations with fundamental arrest characteristics and early physiological markers may remain clinically informative, the findings should be regarded as exploratory and require validation in contemporary multicenter cohorts.

The overall cohort and several clinically important subgroups were small. PE-related outcomes showed near-complete separation, while the lactate and BNP analyses were restricted to patients with available measurements. The BNP landmark analysis excluded patients who died within the first 24 h and therefore estimates only subsequent mortality among early survivors. Although model performance was assessed using bootstrap internal validation, this does not establish external validity or clinical transportability.

Important resuscitation variables, including no-flow time, CPR quality metrics, and time to defibrillation, were not consistently available. Initial pupillary assessment was not standardized and may have influenced resuscitation duration or subsequent treatment decisions, creating the possibility of a self-fulfilling prognostic effect. Arrest etiologies were assigned from clinical documentation and may have been misclassified, while the aggregate category of reversible causes combined heterogeneous conditions. Finally, the targeted secondary analyses and descriptive comparisons were not adjusted for multiplicity. These findings should therefore be regarded as exploratory and require confirmation in contemporary, prospectively characterized multicenter cohorts.

### 4.2. Conclusions

In this retrospective single-center cohort, initial non-shockable rhythm was associated with lower odds of sustained ROSC and 28-day survival and with a higher hazard of 60-day mortality. The presence of a potentially reversible cause was associated with more favorable outcomes, whereas active hematologic malignancy was associated with poorer longer-term survival. Targeted exploratory analyses showed additional associations of lactate, BNP, and initial pupillary findings with subsequent outcomes. However, these results do not establish validated thresholds or prediction tools for individual clinical decisions. Contemporary prospective multicenter studies with standardized data collection and external validation are needed to confirm these associations and assess their potential contribution to IHCA risk stratification.

## Figures and Tables

**Table 1:** Baseline characteristics and clinical parameters of patients with in-hospital cardiac arrest, stratified by ROSC outcome. This table summarizes the baseline demographic data, clinical characteristics, resuscitation parameters, event locations, presumed causes of cardiac arrest, clinical laboratory values, and comorbidities of 134 patients with confirmed in-hospital cardiac arrest (IHCA). Patients were stratified according to the presence or absence of return of spontaneous circulation (ROSC) following cardiopulmonary resuscitation (CPR). Continuous variables are reported as mean ± standard deviation if normally distributed, or as median (interquartile range) if non-normally distributed. Categorical variables are presented as number (percentage). Group comparisons between ROSC and no-ROSC patients were performed using Welch’s *t*-test or Mann–Whitney U test for continuous variables and the chi-square test or Fisher’s exact test for categorical variables, as appropriate. Statistically significant *p*-values (<0.05) are shown in bold. Exact *p*-values are reported to four decimal places; values below 0.0001 and above 0.9999 are presented as <0.0001 and >0.9999, respectively.

Variable	Total (*n* = 134)	ROSC (*n* = 99)	No ROSC (*n* = 35)	*p*-Value
Demographic and general data				
Age, years	64.1 ± 13.9	63.3 ± 13.1	66.4 ± 15.8	0.3057
Male sex	91 (67.9)	66 (66.7)	25 (71.4)	0.6040
Weekend or public holiday	20 (14.9)	16 (16.2)	4 (11.4)	0.4994
Resuscitation characteristics				
Duration of resuscitation, min	45 (30–61.3)	45 (30–60)	40 (30–70)	0.4748
Initial GCS score at CPR-team arrival	3 (3–3)	3 (3–3)	3 (3–3)	0.1179
Initially dilated pupils at CPR-team arrival	52 (38.8)	32 (32.3)	20 (57.1)	**0.0096**
Defibrillation	39 (29.1)	32 (32.3)	7 (20.0)	0.1677
Epinephrine use	108 (80.6)	76 (76.8)	32 (91.4)	0.0594
Total epinephrine dose, mg	4 (1–6)	3 (1–5)	7 (4.3–11.5)	**<0.0001**
Atropine use	40 (29.9)	27 (27.3)	13 (37.1)	0.2727
Amiodarone use	44 (32.8)	36 (36.4)	8 (22.9)	0.1436
Rocuronium use	52 (38.8)	45 (45.5)	7 (20.0)	**0.0079**
Intubation during resuscitation	111 (82.8)	79 (79.8)	32 (91.4)	0.1168
Event witnessed by medical staff	93 (69.4)	75 (75.8)	18 (51.4)	**0.0073**
CPR initiated before arrival of the resuscitation team	129 (96.3)	97 (98.0)	32 (91.4)	0.1116
Event locations				
Ambulatory care unit	21 (15.7)	15 (15.2)	6 (17.1)	0.7806
General ward	47 (35.1)	30 (30.3)	17 (48.6)	0.0516
IMC/ICU/telemetry ward	43 (32.1)	36 (36.4)	7 (20.0)	0.0747
Functional unit	19 (14.2)	15 (15.2)	4 (11.4)	0.7797
On-campus (hospital premises)	4 (3.0)	3 (3.0)	1 (2.9)	>0.9999
Initial rhythm at CPR-team arrival				
Non-shockable rhythm (asystole/PEA)	93 (69.4)	62 (62.6)	31 (88.6)	0.0042
Shockable rhythm (VF/pulseless VT)	27 (20.1)	24 (24.2)	3 (8.6)	0.0470
Sinus rhythm	11 (8.2)	10 (10.1)	1 (2.9)	0.2872
Undefined rhythm	3 (2.2)	3 (3.0)	0 (0.0)	0.5670
Presumed cause of cardiac arrest				
Acute coronary syndrome	31 (23.1)	20 (20.2)	11 (31.4)	0.1758
Cardiac decompensation/heart failure	6 (4.5)	4 (4.0)	2 (5.7)	0.6513
Arrhythmia	5 (3.7)	5 (5.1)	0 (0.0)	0.3258
Sepsis	23 (17.2)	17 (17.2)	6 (17.1)	0.9969
Endocarditis	1 (0.7)	0 (0.0)	1 (2.9)	0.2612
Unclear or multifactorial etiology	19 (14.2)	11 (11.1)	8 (22.9)	0.0974
Any reversible cause ^†^	49 (36.6)	42 (42.4)	7 (20.0)	0.0179
Hypoxia	12 (9.0)	11 (11.1)	1 (2.9)	0.1836
Hypovolemia	12 (9.0)	8 (8.1)	4 (11.4)	0.5111
Hypothermia	2 (1.5)	1 (1.0)	1 (2.9)	0.4556
Hypo-/hyperkalemia	8 (6.0)	8 (8.1)	0 (0.0)	0.1107
Pulmonary embolism (PE)	23 (17.2)	22 (22.2)	1 (2.9)	0.0081
Outcome parameters				
Sustained ROSC > 20 min	91 (67.9)	91 (91.9)	-	-
48-h survival	69 (51.5)	69 (69.7)	-	-
28-day survival	33 (24.6)	33 (33.3)	-	-
60-day survival	30 (22.4)	30 (30.3)	-	-
Post-resuscitation survival duration, days	0.1 (0–3)	2 (0.1–6)	0 (0–0)	-
Clinical and laboratory parameters				
Length of hospital stay prior to cardiac arrest, days	8 (3–16)	8 (3–19)	6 (2.8–15)	0.1899
Mean arterial pressure (MAP) on the day of resuscitation (before cardiac arrest), mmHg	80.9 ± 19.0	81.5 ± 18.2	79.4 ± 21.4	0.6740
Heart rate on the day of resuscitation (before cardiac arrest),/min	88.6 ± 27.6	88.2 ± 24.6	89.2 ± 32.2	0.8853
Lactate level at CPR-team arrival, mmol/L	6.4 (3.3–10.9)	6.2 (3.2–9.9)	10.2 (5.2–20.8)	0.1013
Serum potassium level at CPR-team arrival, mmol/L	4.3 (3.9–4.8)	4.2 (3.8–4.8)	4.4 (3.9–4.8)	0.8181
Glucose level at CPR-team arrival, mg/dL	139 (102–206)	146 (101–202)	134 (100–281)	0.9524
Hemoglobin level at CPR-team arrival, g/dL	9.7 (8.3–11.4)	9.6 (8.4–11.6)	9.7 (8.1–11.2)	0.7941
Comorbidities				
Coronary artery disease (CAD)	61 (45.5)	47 (47.5)	14 (40.0)	0.4453
Arterial hypertension	92 (68.7)	71 (71.7)	21 (60.0)	0.1990
Diabetes mellitus	45 (33.6)	35 (35.4)	10 (28.6)	0.4652
Chronic obstructive pulmonary disease (COPD)	11 (8.2)	8 (8.1)	3 (8.6)	>0.9999
Liver cirrhosis	28 (20.9)	25 (25.3)	3 (8.6)	**0.0369**
Chronic kidney disease (CKD)	24 (17.9)	18 (18.2)	6 (17.1)	0.8904
Active malignancy	42 (31.3)	28 (28.3)	14 (40.0)	0.1990
Active hematologic malignancy	23 (17.2)	14 (14.1)	9 (25.7)	0.1186
Immunosuppression	13 (9.7)	10 (10.1)	3 (8.6)	>0.9999

^†^ “Any reversible cause” was a derived binary aggregate variable comprising hypoxia, hypovolemia, hypothermia, hypo-/hyperkalemia, and pulmonary embolism. The component categories were not mutually exclusive, as eight patients had two presumed reversible causes. Consequently, the component counts sum to 57, whereas the aggregate category includes 49 unique patients with at least one reversible cause, each counted only once. Abbreviations: CPR = cardiopulmonary resuscitation; ROSC = return of spontaneous circulation; IMC = intermediate care; ICU = intensive care unit; VF = ventricular fibrillation; VT = ventricular tachycardia; PEA = pulseless electrical activity; GCS = Glasgow Coma Scale; PE = pulmonary embolism; MAP = mean arterial pressure; CAD = coronary artery disease; COPD = chronic obstructive pulmonary disease; CKD = chronic kidney disease.

**Table 2:** Firth penalized logistic regression analysis of factors associated with sustained ROSC (>20 min) following in-hospital cardiac arrest. 91 of 134 patients achieved sustained ROSC. Univariable and multivariable odds ratios were estimated using Firth’s bias-reduced penalized-likelihood logistic regression. The parsimonious multivariable model included age, non-shockable rhythm at CPR-team arrival, witnessed arrest, and any reversible cause of cardiac arrest, selected on the basis of clinical relevance. Age was modeled continuously per 10-year increase. Binary variables were coded as 1 = yes and 0 = no. The category “any reversible cause” comprised hypoxia, hypovolemia, hypothermia, hypo-/hyperkalemia, or pulmonary embolism. Confidence intervals are profile penalized-likelihood intervals, and *p*-values are based on penalized likelihood-ratio tests. Statistically significant *p*-values (<0.05) are shown in bold.

Variable	Univariable OR (95% CI)	*p*-Value	Adjusted OR (95% CI)	*p*-Value
Age, per 10-year increase	0.85 (0.65–1.11)	0.2488	0.87 (0.65–1.16)	0.3443
Non-shockable rhythm at CPR-team arrival, yes vs. no	0.22 (0.07–0.55)	**0.0008**	0.26 (0.09–0.69)	**0.0056**
Event witnessed by medical staff, yes vs. no	2.44 (1.13–5.32)	**0.0240**	2.03 (0.88–4.70)	0.0972
Any reversible cause of cardiac arrest, yes vs. no	3.26 (1.43–8.08)	**0.0043**	3.18 (1.36–8.14)	**0.0072**

Abbreviations: OR, odds ratio; CI, confidence interval; CPR, cardiopulmonary resuscitation.

**Table 3:** Firth penalized logistic regression analysis of factors associated with 28-day survival following in-hospital cardiac arrest. 33 of 134 patients survived to day 28. Univariable and multivariable odds ratios were estimated using Firth’s bias-reduced penalized-likelihood logistic regression. The parsimonious multivariable model included age, non-shockable rhythm at CPR-team arrival, and any reversible cause of cardiac arrest, selected based on clinical relevance. Age was modeled continuously per 10-year increase. Binary variables were coded as 1 = yes and 0 = no. The category “any reversible cause” comprised hypoxia, hypovolemia, hypothermia, hypo-/hyperkalemia, or pulmonary embolism. Confidence intervals are profile penalized-likelihood intervals, and *p*-values are based on penalized likelihood-ratio tests. Statistically significant *p*-values (<0.05) are shown in bold. Exact *p*-values are reported to four decimal places; values below 0.0001 are presented as <0.0001.

Variable	Univariable OR (95% CI)	*p*-Value	Adjusted OR (95% CI)	*p*-Value
Age, per 10-year increase	0.87 (0.66–1.14)	0.3060	0.89 (0.64–1.23)	0.4652
Non-shockable rhythm at CPR-team arrival, yes vs. no	0.30 (0.13–0.67)	**0.0034**	0.27 (0.10–0.68)	**0.0052**
Any reversible cause of cardiac arrest, yes vs. no	9.49 (4.00–24.53)	**<0.0001**	9.96 (4.05–27.15)	**<0.0001**

Abbreviations: OR, odds ratio; CI, confidence interval; CPR, cardiopulmonary resuscitation.

**Table 4:** Cox proportional hazards regression analysis of factors associated with 60-day mortality following in-hospital cardiac arrest. Analyses included 134 patients, of whom 104 died within 60 days and 30 were alive and administratively censored at day 60. Univariable and multivariable hazard ratios were estimated using Cox proportional hazards regression with the Efron method for tied event times. The parsimonious multivariable model included age, non-shockable rhythm at CPR-team arrival, any reversible cause of cardiac arrest, and active hematologic malignancy, selected based on clinical relevance. Age was modeled continuously per 10-year increase. Binary variables were coded as 1 = yes and 0 = no. The category “any reversible cause” comprised hypoxia, hypovolemia, hypothermia, hypo-/hyperkalemia, or pulmonary embolism. Confidence intervals and coefficient *p*-values are Wald based. Statistically significant *p*-values (<0.05) are shown in bold. Exact *p*-values are reported to four decimal places; values below 0.0001 are presented as <0.0001.

Variable	Univariable HR (95% CI)	*p*-Value	Adjusted HR (95% CI)	*p*-Value
Age, per 10-year increase	1.10 (0.95–1.29)	0.2038	1.09 (0.92–1.28)	0.3226
Non-shockable rhythm at CPR-team arrival, yes vs. no	1.97 (1.27–3.07)	**0.0025**	1.96 (1.25–3.08)	**0.0034**
Any reversible cause of cardiac arrest, yes vs. no	0.28 (0.18–0.45)	**<0.0001**	0.27 (0.17–0.44)	**<0.0001**
Active hematologic malignancy, yes vs. no	2.13 (1.32–3.44)	**0.0020**	1.89 (1.15–3.10)	**0.0115**

Abbreviations: HR, hazard ratio; CI, confidence interval; CPR, cardiopulmonary resuscitation.

## Data Availability

The original contributions presented in this study are included in the article and Supplementary Material. Further inquiries can be directed to the corresponding author.

## Supplementary Materials

The following supporting information can be downloaded at: https://www.mdpi.com/article/10.3390/medsci14040480/s1.

## Abbreviations

AHA	American Heart Association
AUC	Area Under the Receiver Operating Characteristic Curve
BNP	B-type Natriuretic Peptide
CI	Confidence Interval
CPR	Cardiopulmonary Resuscitation
ERC	European Resuscitation Council
GCS	Glasgow Coma Scale
GWTG-R	Get With The Guidelines–Resuscitation
HR	Hazard Ratio
IHCA	In-Hospital Cardiac Arrest
OR	Odds Ratio
PE	Pulmonary Embolism
PEA	Pulseless Electrical Activity
ROC	Receiver Operating Characteristic
ROSC	Return of Spontaneous Circulation
SD	Standard Deviation
VF	Ventricular Fibrillation
VT	Ventricular Tachycardia
