# Supplementary material for: Predictors and Outcomes of In-Hospital Cardiac Arrest in University Medicine with Advanced Tertiary and Transplant Care
Source: Med Sci (Basel). 2026 Aug 14;14(4):480. doi: 10.3390/medsci14040480 (PMC13515621; doi:10.3390/medsci14040480)
Supplement: Supplementary file 1 [file medsci-14-00480-s001.zip › medsci-4423149-supplementary.pdf]

Supplementary Table S1. Targeted secondary Firth penalized logistic regression analyses of selected factors associated with 28-day survival following in-hospital cardiac arrest. Both analyses included all 134 patients, of whom 33 survived to day 28 and 101 did not. Model A extended the primary 28-day survival model by active hematologic malignancy, whereas Model B extended it by initially dilated pupils at CPR-team arrival. Each model included age, non-shockable rhythm at CPR-team arrival, any reversible cause of cardiac arrest, and one targeted exploratory factor. Age was modeled continuously per 10-year increase. Binary variables were coded as 1 = yes and 0 = no. The category "any reversible cause" comprised hypoxia, hypovolemia, hypothermia, hypo-/hyperkalemia, or pulmonary embolism. Odds ratios were estimated using Firth's bias-reduced penalized-likelihood logistic regression. Confidence intervals are profile penalized-likelihood intervals, and coefficient p-values are based on penalized likelihood-ratio tests. Apparent and optimism-corrected AUCs were estimated using 1,000 bootstrap resamples. These targeted secondary analyses were exploratory. Statistically significant p-values (< 0.05) are shown in bold. Exact p-values are reported to four decimal places; values below 0.0001 are presented as <0.0001. Abbreviations: OR, odds ratio; CI, confidence interval; CPR, cardiopulmonary resuscitation; AUC, area under the receiver operating characteristic curve.

Model A: Primary model extended by active hematologic malignancy

Variable	Univariable OR (95% CI)	p-value	Adjusted OR (95% CI)	p-value
Age, per 10-year increase	0.87 (0.66–1.14)	0.3060	0.88 (0.63–1.22)	0.4331
Non-shockable rhythm at CPR-team arrival, yes vs. no	0.30 (0.13–0.67)	**0.0034**	0.29 (0.11–0.75)	**0.0101**
Any reversible cause of cardiac arrest, yes vs. no	9.49 (4.00–24.53)	**<0.0001**	9.72 (3.90–26.83)	**<0.0001**
Active hematologic malignancy, yes vs. no	0.16 (0.02–0.68)	**0.0090**	0.18 (0.02–0.91)	**0.0367**

Model performance: penalized likelihood-ratio $\chi^2 = 40.41$, df = 4, $p < 0.0001$; apparent AUC = 0.859 (95% bootstrap CI 0.794–0.923); optimism-corrected AUC = 0.840 (95% location-shifted bootstrap CI 0.775–0.904); apparent and optimism-corrected Brier scores = 0.130 and 0.143.

Model B: Primary model extended by initially dilated pupils

Variable	Univariable OR (95% CI)	p-value	Adjusted OR (95% CI)	p-value
Age, per 10-year increase	0.87 (0.66–1.14)	0.3060	0.79 (0.54–1.13)	0.2009

Non-shockable rhythm at CPR-team arrival, yes vs. no	0.30 (0.13–0.67)	**0.0034**	0.31 (0.11–0.84)	**0.0212**
Any reversible cause of cardiac arrest, yes vs. no	9.49 (4.00–24.53)	**<0.0001**	9.55 (3.58–29.01)	**<0.0001**
Initially dilated pupils at CPR-team arrival, yes vs. no	0.08 (0.02–0.26)	**<0.0001**	0.09 (0.01–0.33)	**0.0001**

Model performance: penalized likelihood-ratio $\chi^2 = 51.04$, df = 4, $p < 0.0001$; apparent AUC = 0.885 (95% bootstrap CI 0.826–0.950); optimism-corrected AUC = 0.871 (95% location-shifted bootstrap CI 0.812–0.936); apparent and optimism-corrected Brier scores = 0.111 and 0.123.

Supplementary Table S2. Cox proportional hazards analysis of lactate concentration and 60-day mortality following in-hospital cardiac arrest. The analysis included 91 patients with an available lactate measurement at CPR-team arrival, of whom 65 died within 60 days and 26 were alive and administratively censored at day 60. Univariable and multivariable hazard ratios were estimated using Cox proportional hazards regression with Efron handling of tied event times. The targeted biomarker model included age, non-shockable rhythm at CPR-team arrival, any reversible cause of cardiac arrest, active hematologic malignancy, and lactate concentration, selected based on clinical relevance. Age was modeled continuously per 10-year increase, and lactate was modeled continuously per 1 mmol/L increase. Binary variables were coded as 1 = yes and 0 = no. The category "any reversible cause" comprised hypoxia, hypovolemia, hypothermia, hypo-/hyperkalemia, or pulmonary embolism. Hazard-ratio confidence intervals and coefficient p-values are Wald based. Apparent and optimism-corrected performance estimates were obtained by bootstrap internal validation. Because lactate was unavailable in 43 patients, this complete-case biomarker analysis was considered exploratory. Statistically significant p-values (< 0.05) are shown in bold. Exact p-values are reported to four decimal places; values below 0.0001 are presented as <0.0001. Abbreviations: HR, hazard ratio; CI, confidence interval; CPR, cardiopulmonary resuscitation.

Variable	Univariable HR (95% CI)	p-value	Adjusted HR (95% CI)	p-value
Age, per 10-year increase	1.09 (0.89–1.33)	0.3859	1.10 (0.90–1.36)	0.3590
Non-shockable rhythm at CPR-team arrival, yes vs. no	1.85 (1.08–3.16)	**0.0252**	1.96 (1.11–3.45)	**0.0201**
Any reversible cause of cardiac arrest, yes vs. no	0.29 (0.16–0.52)	**<0.0001**	0.41 (0.23–0.75)	**0.0039**
Active hematologic malignancy, yes vs. no	2.44 (1.19–4.98)	**0.0145**	1.92 (0.90–4.08)	0.0912
Lactate at CPR-team arrival, per 1 mmol/L increase	1.14 (1.09–1.18)	**<0.0001**	1.12 (1.07–1.17)	**<0.0001**

Model performance: likelihood-ratio $\chi^2 = 54.18$, df = 5, $p < 0.0001$; apparent Harrell C-index = 0.796 (95% bootstrap CI 0.735–0.861); optimism-corrected Harrell C-index = 0.780 (95% location-shifted bootstrap CI 0.719–0.845); apparent and optimism-corrected 60-day Brier scores = 0.120 and 0.132, respectively.

Supplementary Table S3. Twenty-four-hour landmark Cox proportional hazards analysis of B-type natriuretic peptide and subsequent mortality through day 60 following in-hospital cardiac arrest. The landmark analysis included 55 patients who were alive beyond 24 hours and had a BNP measurement obtained within the first 24 hours after resuscitation; 31 died subsequently, while 24 were alive and administratively censored at day 60. Follow-up was reset at 24 hours and continued through day 60. Univariable and multivariable hazard ratios were estimated using Cox proportional hazards regression with Efron handling of tied event times. The targeted model included age, non-shockable rhythm at CPR-team arrival, any reversible cause of cardiac arrest, active hematologic malignancy, and log_2-transformed BNP. BNP effects are expressed per doubling. Age was modeled continuously per 10-year increase. Binary variables were coded as 1 = yes and 0 = no. The category "any reversible cause" comprised hypoxia, hypovolemia, hypothermia, hypo-/hyperkalemia, or pulmonary embolism. Hazard-ratio confidence intervals and coefficient p-values are Wald based. Apparent and optimism-corrected performance estimates were obtained using 1,000 bootstrap resamples. In a reduced sensitivity model excluding active hematologic malignancy, the BNP association remained materially unchanged (adjusted HR = 1.30, 95% CI 1.08–1.57, p = 0.0059). Because BNP was available only in a selected subgroup and five predictors were evaluated with 31 post-landmark deaths, this analysis was considered exploratory. Statistically significant p-values (< 0.05) are shown in bold. Abbreviations: HR, hazard ratio; CI, confidence interval; CPR, cardiopulmonary resuscitation; BNP, B-type natriuretic peptide.

Variable	Univariable HR (95% CI)	p-value	Adjusted HR (95% CI)	p-value
Age, per 10-year increase	1.00 (0.75–1.34)	0.9861	0.84 (0.60–1.17)	0.2996
Non-shockable rhythm at CPR-team arrival, yes vs. no	1.40 (0.68–2.89)	0.3596	1.03 (0.46–2.29)	0.9430
Any reversible cause of cardiac arrest, yes vs. no	0.23 (0.10–0.50)	**0.0002**	0.21 (0.09–0.46)	**0.0001**
Active hematologic malignancy, yes vs. no	2.46 (0.85–7.11)	0.0950	3.98 (1.21–13.04)	**0.0227**
BNP measured within 24 hours, per doubling	1.26 (1.07–1.49)	**0.0055**	1.31 (1.09–1.59)	**0.0049**

Model performance: likelihood-ratio χ^2 = 30.01, df = 5, p < 0.0001; apparent Harrell C-index = 0.775 (95% bootstrap CI 0.692–0.876); optimism-corrected Harrell C-index = 0.743 (95% location-shifted bootstrap CI 0.659–0.844); apparent and optimism-corrected day-60 Brier scores = 0.145 and 0.172, respectively.

Supplementary Table S4. Exploratory unadjusted outcomes according to pulmonary embolism-related cardiac arrest. Outcomes were compared between patients with pulmonary embolism (PE)-related in-hospital cardiac arrest and patients with all other presumed arrest etiologies. Absolute risk differences were calculated as the outcome proportion in the PE group minus the corresponding proportion in the other-etiology group; 95% confidence intervals were calculated using the Newcombe–Wilson method without continuity correction. Two-sided Fisher's exact tests were used because of the small PE subgroup and sparse cells. P-values were not adjusted for multiple comparisons and should be interpreted as exploratory. Because the PE subgroup was small and several outcomes showed near-complete separation, no PE-specific multivariable regression model was fitted. PE was retained only as a component of the aggregate variable "any reversible cause" in the primary multivariable models. Statistically significant p-values (< 0.05) are shown in bold. Exact p-values are reported to four decimal places; values below 0.0001 are presented as <0.0001. Abbreviations: PE, pulmonary embolism; IHCA, in-hospital cardiac arrest; ROSC, return of spontaneous circulation; CI, confidence interval.

Outcome	PE-related IHCA, n/N (%)	Other etiologies, n/N (%)	Absolute risk difference, percentage points (95% CI)	p-value
Any ROSC	22/23 (95.7%)	77/111 (69.4%)	26.3 (7.9–36.1)	**0.0081**
Sustained ROSC >20 min	22/23 (95.7%)	69/111 (62.2%)	33.5 (14.8–43.4)	**0.0011**
48-hour survival	22/23 (95.7%)	47/111 (42.3%)	53.3 (34.2–62.8)	**<0.0001**
28-day survival	22/23 (95.7%)	11/111 (9.9%)	85.7 (67.7–91.3)	**<0.0001**
60-day survival	22/23 (95.7%)	8/111 (7.2%)	88.4 (70.6–93.5)	**<0.0001**

Supplementary Figure S1.

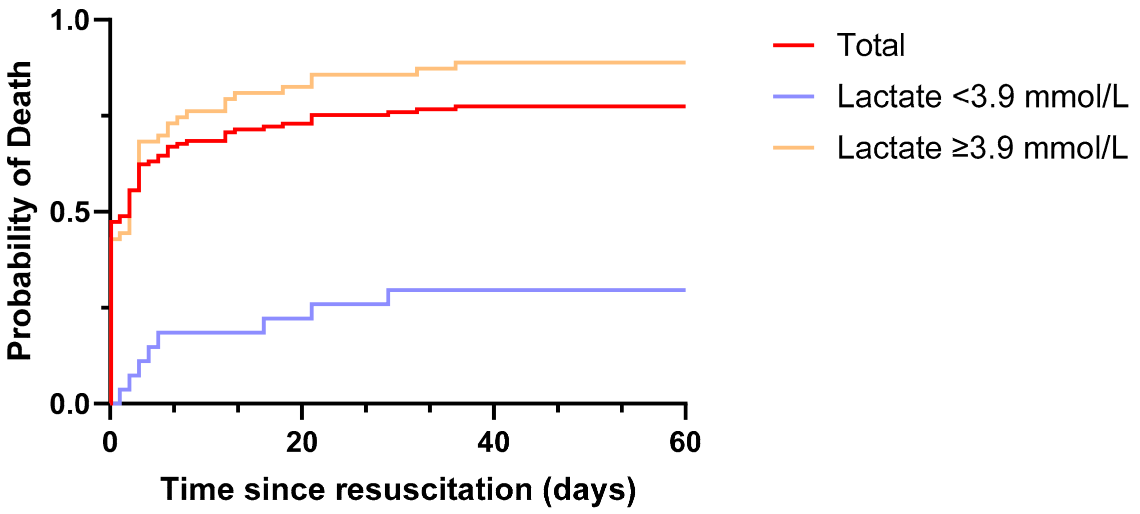

Supplementary Figure S1. Kaplan–Meier estimates of cumulative 60-day mortality after in-hospital cardiac arrest, stratified by lactate concentration at CPR-team arrival.

The curves depict the cumulative probability of death, calculated as 1 minus the Kaplan–Meier survival estimate, from the onset of cardiopulmonary resuscitation through day 60. The red curve represents the entire cohort (n = 134) and is shown for descriptive reference. Patients with available lactate measurements (n = 91) were stratified according to the exploratory receiver operating characteristic-derived cut-off for 60-day mortality: lactate <3.9 mmol/L (n = 27) versus lactate ≥3.9 mmol/L (n = 64). The two lactate groups were compared using the two-sided log-rank (Mantel–Cox) test ($p < 0.0001$). Because the threshold was derived and evaluated in the same cohort, this analysis is exploratory and may overestimate the discriminatory performance of the cut-off.
